# Effect of Dietary Iron Loading on Recognition Memory in Growing Rats

**DOI:** 10.1371/journal.pone.0120609

**Published:** 2015-03-06

**Authors:** Murui Han, Jonghan Kim

**Affiliations:** Department of Pharmaceutical Sciences, Northeastern University, Boston, Massachusetts, United States of America; University of Victoria, CANADA

## Abstract

While nutritional and neurobehavioral problems are associated with both iron deficiency during growth and overload in the elderly, the effect of iron loading in growing ages on neurobehavioral performance has not been fully explored. To characterize the role of dietary iron loading in memory function in the young, weanling rats were fed iron-loading diet (10,000 mg iron/kg diet) or iron-adequate control diet (50 mg/kg) for one month, during which a battery of behavioral tests were conducted. Iron-loaded rats displayed elevated non-heme iron levels in serum and liver, indicating a condition of systemic iron overload. In the brain, non-heme iron was elevated in the prefrontal cortex of iron-loaded rats compared with controls, whereas there was no difference in iron content in other brain regions between the two diet groups. While iron loading did not alter motor coordination or anxiety-like behavior, iron-loaded rats exhibited a better recognition memory, as represented by an increased novel object recognition index (22% increase from the reference value) than control rats (12% increase; P=0.047). Western blot analysis showed an up-regulation of dopamine receptor 1 in the prefrontal cortex from iron-loaded rats (142% increase; P=0.002). Furthermore, levels of glutamate receptors (both NMDA and AMPA) and nicotinic acetylcholine receptor (nAChR) were significantly elevated in the prefrontal cortex of iron-loaded rats (62% increase in NR1; 70% increase in Glu1A; 115% increase in nAChR). Dietary iron loading also increased the expression of NMDA receptors and nAChR in the hippocampus. These results support the idea that iron is essential for learning and memory and further reveal that iron supplementation during developmental and rapidly growing periods of life improves memory performance. Our investigation also demonstrates that both cholinergic and glutamatergic neurotransmission pathways are regulated by dietary iron and provides a molecular basis for the role of iron loading in improved memory.

## Introduction

A strong relationship exists between iron status and neurobehavioral functions [1–8]. Iron is essential for the development and proper function of the brain, including myelination [9], monoamine metabolism [10] and regulation of nitric oxide synthase [11]. Iron is a critical cofactor for tyrosine hydroxylase and tryptophan hydroxylase, which are enzymes for dopamine and serotonin synthesis, respectively. Iron also regulates homeostasis of glutamate and γ-aminobutyric acid (GABA) [12]. Moreover, a recent investigation has demonstrated that iron supplementation enhances brain synaptic plasticity by activation of N-methyl-D-aspartate (NMDA) receptor, a receptor associated with memory function [13], suggesting that altered iron status in the brain significantly modulates neurotransmission pathways and neural activities.

Iron deficiency leads to abnormal cognitive function and behavioral deficits, especially in the early stage of life. For example, Lozoff *et al* have noted that the formerly iron-deficient children exhibit reduced visual-spatial memory function and delays in cognitive processing even though their anemic status was corrected later by iron therapy [14]. Diminished mental and motor development is associated with iron deficiency in infants [15]. In rats, a period of rapid growth occurs in the first 2–3 weeks of postnatal life [16,17], in which iron demand is very high. Consequently, iron transport into the brain at this stage is dramatically increased through the blood-brain barrier (BBB) *via* transferrin receptor-mediated uptake [18,19], whereas adult rats display slow rates of iron uptake into the brain. Within the brain, iron is particularly concentrated in the basal ganglia, an area highly influenced by dopamine metabolism [10,20,21]. In addition to its region-specific distribution, there is a prioritization of brain iron distribution during development [22,23]. For instance, after a short period of feeding a low-iron diet, iron stores significantly decrease in the cortex and striatum during the mid-late neonatal periods in rodents (equivalent to human ages 6–12 months), but not in the thalamus, which becomes more sensitive to dietary iron during postweaning iron deficiency [15].

In contrast to iron deficiency, iron accumulation has been implicated in elevated oxidative stress and in the development of age-related neurodegenerative diseases [24–28]. Brain iron levels increase with age [1,29,30]; this has been shown to occur mainly in brain regions that are affected by the disease states, including Alzheimer’s, Parkinson’s, and Huntington’s diseases [30]. Iron overload also disrupts neurotransmitter homeostasis. For example, iron infusions into the substantia nigra impair monoaminergic systems, especially the dopaminergic pathway, to promote motor function deficits resembling Parkinson’s disease [31–33]. The effects of iron overload on learning and memory deficits have been noted in animals [34–38]. Likewise, iron overload appears to alter anxiety-like behavior and mood [39,40]. Anxious responses, determined by the elevated plus maze, are observed in adult rats receiving daily intraperitoneal injections of iron [40]. Other behavioral impairments have been found in rats fed diet containing 20,000 ppm iron for 12 weeks [39].

While both iron deficiency during growth and overload in the elderly produce neurobehavioral problems, the effect of iron loading in postweaning ages on neurobehavioral performance has not been fully explored. Thus, we here investigated the role of dietary iron loading in cognitive and behavioral function using weanling rats. We found that rats fed high iron diet improved recognition memory, which was associated with elevated expression of dopaminergic, glutamatergic and cholinergic receptors in the prefrontal cortex and hippocampus. Our investigation suggests that dietary iron loading for a short-term period augments memory function during developmental and rapidly growing periods of life.

## Materials and Methods

### Animals and diets

Animal protocols were approved by the Division of Laboratory Animal Medicine (DLAM) and the Northeastern University-Institutional Animal Care and Use Committee (NU-IACUC). Weanling male Sprague-Dawley (SD) rats were purchased from Charles River Laboratories (Wilmington, MA, USA) and assigned into two groups according to the ranked body weight. Male rats were used because estrogen affects iron metabolism [41,42]. Rats were maintained on a 12:12-h light-dark cycle and given water *ad libitum* provided by DLAM. Rats were fed either iron-loading diet containing 10,000 mg iron/kg (as carbonyl iron; TD.09077, Harlan Teklad, Madison, WI, USA) or iron-adequate control diet containing 50 mg iron/kg (TD.07800, Harlan Teklad) for a total of one month (30 days). The iron-loading diet model has previously been used to investigate the role of dietary iron overload in altered physiology [43,44]. During the diet period, a battery of behavioral tests (elevated plus maze, novel object recognition task and rotarod) were conducted.

### Elevated plus maze test

Animal behavior for anxiety and impulsivity was tested based on the previously reported method [45] using an elevated plus maze (Harvard Apparatus, Holliston, MA, USA) which consisted of two open arms and two closed arms. At the age of 6 weeks (e.g., after feeding iron diet for 3 weeks), each rat was placed in the center of the maze, facing one of the open arms, and allowed to explore the maze for 5 min. The test area was enclosed by curtains with dim light. The latency of the first entry into an open arm, number of entries and time spent in the center, open and closed arms, as well as total distance traveled, were recorded by a CMOS camera and analyzed by ANY-maze software (Stoelting Co., Wood Dale, IL, USA), which detected the center point of each animal. The apparatus was cleaned with Quatricide TB (Pharmacal Research Laboratories, Naugatuck, CT, USA) between each animal test.

### Novel object recognition task

One day after elevated plus maze test, rats were acclimated to the open field (Harvard Apparatus; 122 L x 122 W x 41 H cm) with two identical familiar objects (objects 1A and 2A) for 5 min per trial, twice a day with 2 h interval over 2 days prior to the novel object recognition test. For the recognition memory test, in the first trial the two identical objects (1A and 2A) used during the acclimation period were positioned symmetrically in the open field. A rat was placed in the center of the open field, allowed to explore freely for 5 min and then returned to home cage. To avoid olfactory cues, the open field and the objects were cleaned with Quatricide TB thoroughly between the trials. After 2 h, the rat was placed again for another 5 min in the open field, in which one of the familiar objects (A) was replaced by a novel object (B) with different shape and color. The time spent with both novel and familiar objects (head-tracking) and total distance traveled (center point-tracking) during the session were recorded and analyzed by ANY-maze. Exploration with the objects was defined as the rat nose was within 2 cm toward the object [46]. The percentage of time spent with the novel object to the total interaction time (time spent with both novel and familiar objects) was defined as recognition index.

### Rotarod test

Effect of iron loading on motor coordination was assessed with a standard accelerating rotarod device (Harvard Apparatus), which consisted of a motor-driven knurled nylon cylinder 6 cm in diameter mounted horizontally 35.5 cm above a padded surface [47]. At the age of 6.5-week, rats were trained on the rotarod with three sessions per day, 3 min/trial, with 5-min inter-trial intervals, at fixed speeds after habituation on the stationary rotarod for 1 min. Day 1 training speeds were 4, 7 and 10 rpm; day 2 training speeds were 7, 10 and 13 rpm; day 3 training speeds were 10, 13 and 16 rpm. On day 4, rats were tested on the rotarod with accelerating speed from 4 rpm to 40 rpm over 5 min or until rats fell off. Each rat was tested on the rotarod twice with a 10-min inter-trial interval. The rotarod device was cleaned using Quatricide TB between trials. Time on bar and speed attained on rotarod before falling were recorded, and the better performance of the two trials was used for analysis [47].

### Tissue collection

Immediately after the rotarod test (7-week-old), rats were euthanized by isofluane overdose, followed by exsanguination and tissue collection, including blood, liver and brain. Serum was harvested from blood. The brain was further microdissected to harvest prefrontal cortex (PFC), striatum, hippocampus (HPC) and cerebellum. For non-heme iron analysis in the brain, a different cohort of weanling rats fed either diet was euthanized at the age of 7-week. All tissues were flash-frozen in liquid nitrogen and stored at -80°C until analysis.

### Western blot analysis

PFC and HPC, the two well-established areas for memory function [48], were homogenized and samples (50 μg proteins) were electrophoresed on a 10% SDS-polyacrylamide gel and transferred to either polyvinylidene difluoride (Millipore, Billerica, MA, USA) or nitrocellulose (GE Healthcare, Piscataway, NJ, USA) membrane. After blocking with 5% non-fat milk, the membrane was incubated in rabbit antibody against α7 nicotinic acetylcholine receptor (nAChR, 1:800; Abcam, Cambridge, MA, USA), α-Amino-3-hydroxy-5-methyl-4-isoxazolepropionic acid (AMPA) glutamate receptor 1 (GluA1, 1:600, Abcam, USA) or dopamine receptor D1 (D1R; 1:500; Abcam) or in goat antibody against NMDA glutamate receptor ζ1 (NR1, 1:100; Santa Cruz Biotech, Dallas, TX, USA), NMDA glutamate receptor ɛ1 (NR2A, 1:100; Santa Cruz Biotech), NMDA glutamate receptor ɛ2 (NR2B, 1:100; Santa Cruz Biotech), AMPA glutamate receptor 2 (GluA2, 1:1,000; Millipore) or dopamine transporter (DAT, 1:100; Santa Cruz Biotech). As a loading control, the immunoblot was incubated with mouse anti-actin (1:5,000; MP Biomedicals, Solon, OH, USA). The blots were incubated with donkey anti-rabbit secondary antibody conjugated with HRP (1:1,000; GE Healthcare), donkey anti-goat antibody (1:1,000; Santa Cruz Biotech) or sheep anti-mouse antibody (1:5,000; GE Healthcare), followed by chemiluminescence (ECL West Dura, Thermo Scientific, Waltham, MA, USA) and scanned using Chemidoc System (ChemiDoc XRS, Bio-Rad, Hercules, CA, USA). Relative intensities of protein bands normalized to actin were determined using Image Lab (Bio-Rad, version 4.1).

### Analysis of malondialdehyde and superoxide dismutase activity

PFC and HPC samples were homogenized using Tris buffer (100 mM, pH 7.4; 10-time dilution). The lysate was split into two aliquots. One aliquot was centrifuged at 1,500 *g* for 5 min at 4°C to measure the activities of superoxide dismutase (SOD) using assay reagents (Cayman Chemical, Ann Arbor, MI, USA) according to manufacturer’s instructions. The other was centrifuged at 10,000 *g* for 15 min at 4°C to measure the levels of malondialdehyde (MDA) using assay reagents (Cayman Chemical). Protein concentration was determined by Pierce BCA Protein Assay Kit (Thermo Scientific). SOD activities were presented as unit per mg protein. The levels of MDA were expressed as μmol/mg protein.

### Analysis of iron status

Tissues (liver and microdissected brain) were incubated in 5–20 fold volume of acid solution (10% trichloroacetic acid, 3 M HCl) in 65°C water bath for 20 h. Non-heme iron concentrations were measured as previously described [49,50] and presented as nmol iron per g of wet tissue weight (nmol/g tissue). Serum iron was determined as previously described with background correction [51] and calculated as nmol/ml in serum.

### Statistical analysis

Values reported were expressed as means ± SEM. Comparisons between iron-loaded and control rats were performed by the Student’s *t*-test. Differences were considered significant at *P* < 0.05.

## Results

### Dietary iron loading modifies systemic and brain iron status in growing rats

**Fig 1:**
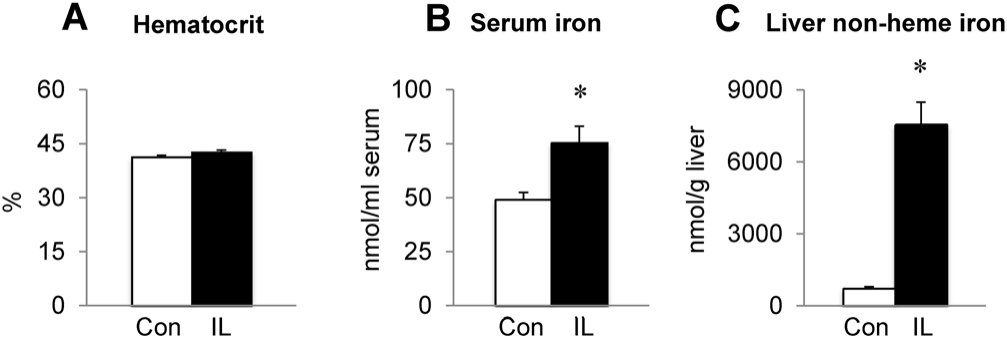
Effect of dietary iron loading on systemic iron status in growing rats. Weanling rats were fed control (50 mg iron/kg diet) or iron-loading diet (10,000 mg iron as carbonyl iron per kg diet) for one month and euthanized to collect blood, serum and liver. For systemic iron status, hematocrit (**A**) and the levels of serum iron (**B**) and liver non-heme iron (**C**) were determined. Empty and closed bars represent control (Con) and iron-loaded (IL) rats, respectively. Data were presented as means ± SEM (n = 6 per group) and were analyzed using two-sample *t*-test. * *P* < 0.05.

Weanling male rats were fed control or iron loading (10,000 mg iron/kg) diet over a month. Iron loading diet decreased body weight (274 ± 8 g; n = 12) compared with controls (318 ± 10 g; n = 16; *P* = 0.004), although total food intake per body weight was not different (data not shown). Decreased body weight upon iron loading was previously reported in rats fed 3,500 and 20,000 ppm iron [39]. While hematocrit values were unchanged upon iron loading (Fig. 1A), iron-loaded rats displayed significantly elevated levels of iron in serum (Fig. 1B) and liver (Fig. 1C), indicating a condition of systemic iron loading due to increased dietary iron. To examine if dietary iron loading in growing rats also increases iron stores in the brain, the left brain hemisphere from rats fed either control or iron loading diet was microdissected for iron analysis. Non-heme iron levels in the PFC were significantly elevated in iron-loaded rats compared with controls (*P* = 0.018; n = 5–6/group), while those in other regions (i.e., striatum, hippocampus and cerebellum) did not significantly differ between the two groups (Table 1).

**Table 1:** Effect of dietary iron loading on brain iron status in growing rats.

Brain region	Control rats	Iron-loaded rats	*P* value
	(nmol/g brain)		
Prefrontal cortex	168 ± 6	224 ± 18	0.009
Striatum	167 ± 11	127 ± 10	0.157
Hippocampus	134 ± 7	180 ± 20	0.062
Cerebellum	170 ± 5	194 ± 10	0.059

Rats were fed control (50 mg iron/kg diet) or iron-loading diet (10,000 mg iron as carbonyl iron per kg diet) for one month and euthanized. Brain samples were microdissected to quantify iron content in different brain regions by non-heme iron analysis. Data were presented as means ± SEM (n = 5–6 per group) and were analyzed using two-sample *t*-test.

### Neither motor coordination nor anxiety-like behavior is altered upon dietary iron loading in rats

**Fig 2:**
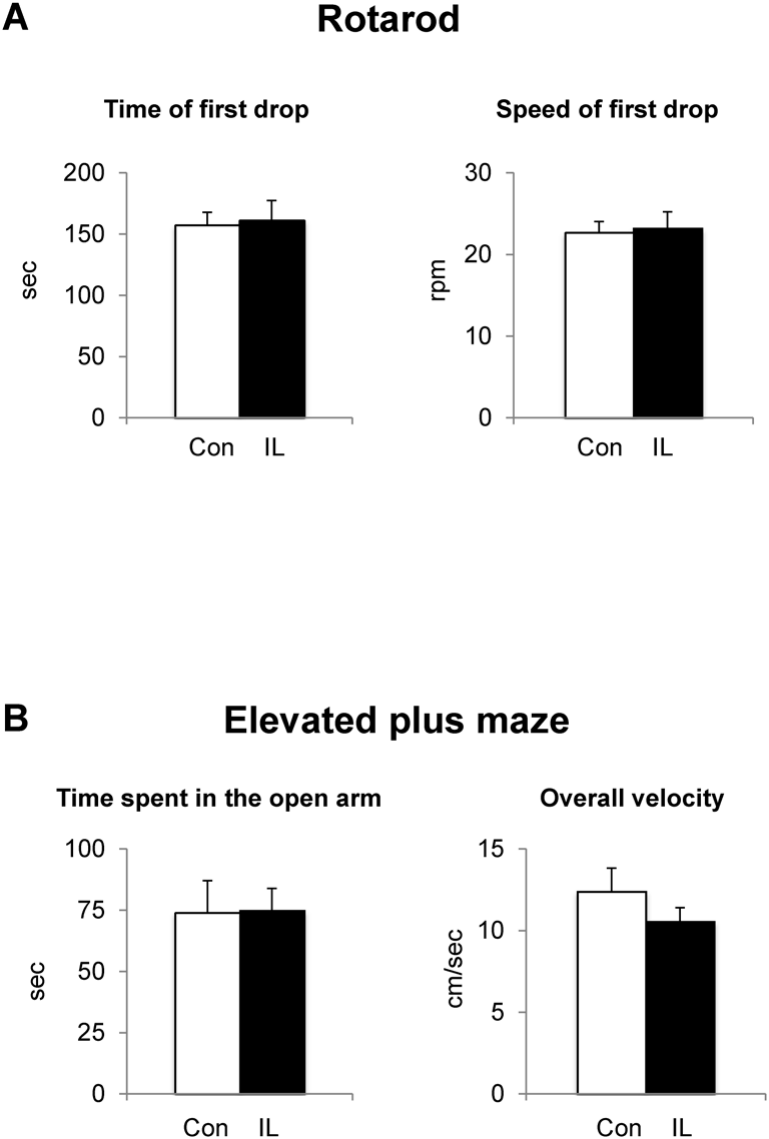
Effect of dietary iron loading on motor coordination and anxiety-like behavior in growing rats. Rats fed control or iron-loading diet for one month were tested on the rotarod device to record the time to falling-off and speed of the rod (**A**) and on the elevated plus maze in order to determine anxiety- and impulsivity-related behavior, including time in open arms and overall velocity in the maze (**B**). Empty and closed bars represent control and iron-loaded rats, respectively. Data were presented as means ± SEM (n = 11 per group) and were analyzed using two-sample *t*-test. * *P* < 0.05.

Since altered brain iron is associated with deficits in motor function [31–33], we determined the effect of dietary iron loading on motor coordination by an accelerating rotarod device. Iron-loaded rats fell off the rotarod about 3 min since the start, which was similar to the performance by control rats (Fig. 2A). The peak speed attained on the rotarod by iron-loaded rats was comparable to that by control rats. Since there is a growing body of evidence that impaired iron metabolism is linked to emotional changes [39,40,52], we investigated if dietary iron overload can alter anxiety and/or impulsivity by the elevated plus maze paradigm. Time spent in open arms or overall velocity in the maze was not different between the two groups (Fig. 2B). Iron-loaded rats spent similar time entering an open arm with the number of entries to open and closed arms unchanged (data not shown). Combined, these results suggest that dietary iron loading does not alter motor coordination or emotional behavior in growing rats.

### Growing rats fed iron loading diet increase recognition memory

**Fig 3:**
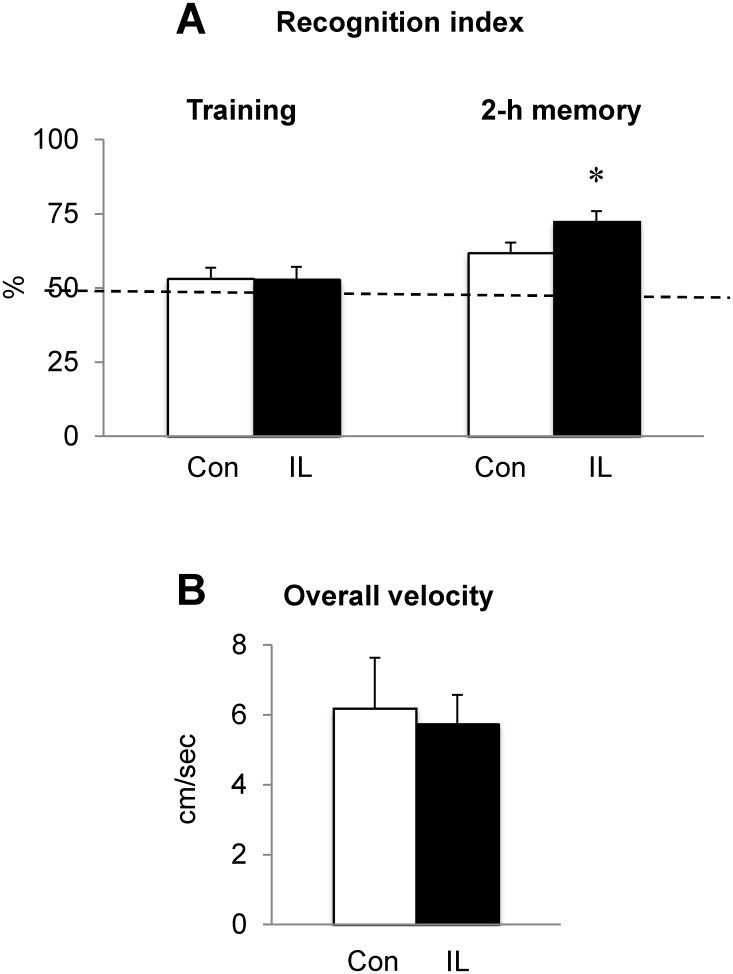
Effect of dietary iron loading on recognition memory in growing rats. Rats fed iron-loading or control diet were subject to the novel object recognition task in an open field where two identical objects were placed. Rat behavior was recorded for 5 min and time of interaction with each object was recorded to quantify recognition index during the training period (**A**). Following 2 h of training with two identical objects, one object was replaced with a different object and time of interaction with each object was recorded to calculate % recognition index (= interaction time with the novel object divided by interaction time with both novel and familiar objects) (**A**). Total velocity traveled in the field was also recorded (**B**). The dotted line represents a reference baseline of 50% random chance. Empty and closed bars represent control (Con) and iron-loaded (IL) rats, respectively. Data were presented as means ± SEM (n = 11 per group) and were analyzed using two-sample *t*-test. * *P* < 0.05.

To evaluate if iron loading influences memory capacity in growing rats, rats fed either high iron or control diet were tested for the novel recognition memory task. During the training session, both control and iron-loaded rats spent similar time in recognizing two identical objects, indicating there was no preference for object location (Fig. 3A). When one of the familiar objects was replaced by a novel object 2 h after the training session, control rats recognized the novel object (61.7%) better than the familiar object (38.3%). Notably, the percent time spent with the novel object increased significantly in iron-loaded rats (72.4%) compared with the familiar object (27.6%). When assessed against 50% random chance, recognition index was significantly increased in iron-loaded rats compared with controls (22.4 *vs* 11.7% from the reference baseline of 50%; *P* = 0.047; n = 11/group; Fig. 3A), indicating an improved recognition memory upon iron loading in growing rats. Overall velocity in the open field was not altered in iron-loaded rats (Fig. 3B), suggesting that the difference in recognition memory was not due to altered exploratory behavior during the test.

### Dietary iron loading up-regulates dopamine D1 receptor levels in PFC

**Fig 4:**
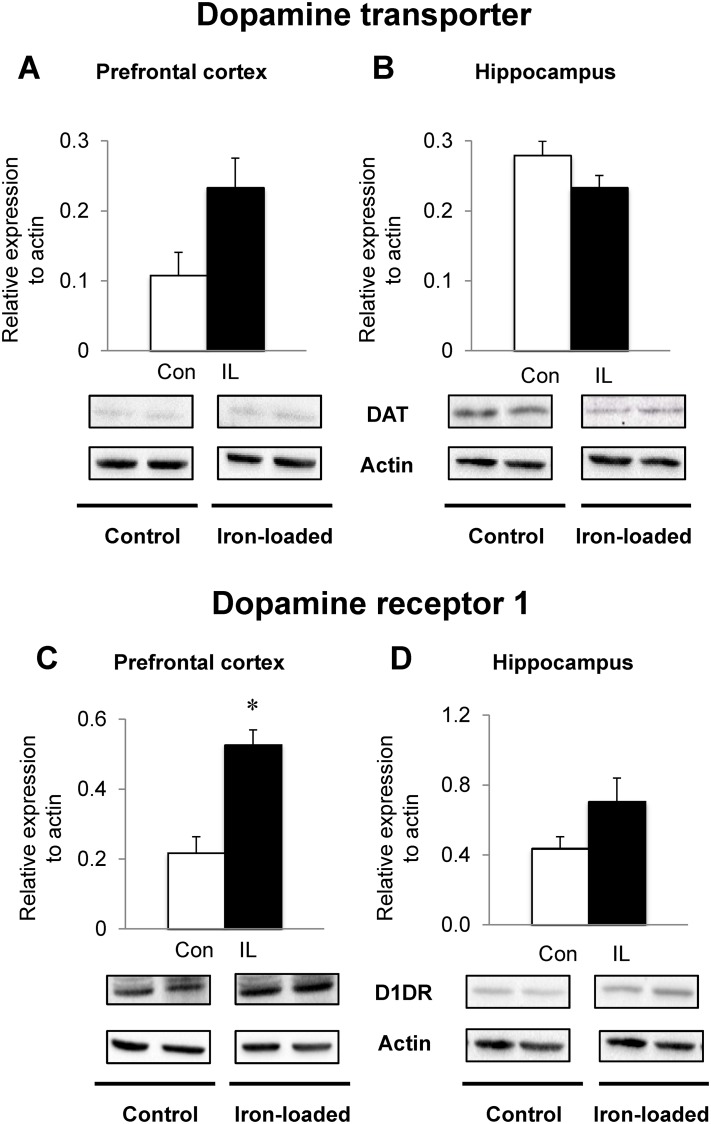
Effect of dietary iron loading on the expression of dopamine transporters and dopamine receptors in young rat brain. Prefrontal cortex (**A and C**) and hippocampus (**B and D**) were collected from rats fed iron-loading or control diet and homogenized for western blot analysis to determine the expression levels of dopamine transporter (**A and B**) or dopamine D1 receptor (**C and D**). Relative intensities of protein bands normalized to actin were determined using Image Lab (version 4.1). Empty and closed bars represent control (Con) and iron-loaded (IL) rats, respectively. Data were presented as means ± SEM (n = 4 per group) and were analyzed using two-sample *t*-test. * *P* < 0.05.

Since dopamine plays an important role in learning capacity and since D1R has been shown to modulate working memory-related neural activity [53], we quantified DAT and D1R in the PFC and HPC. There was no significant difference in DAT levels in either PFC or HPC between two groups of rats (Fig. 4, A and B). In contrast, iron-loaded rats up-regulated D1R protein levels in the PFC compared with controls (142% increase; *P* = 0.002; Fig. 4C). The expression levels of D1R in the HPC from iron-loaded rats trended higher (*P* = 0.089; Fig. 4D), although statistically insignificant.

### Dietary iron loading up-regulates glutamate and acetylcholine receptors in PFC and HPC

**Fig 5:**
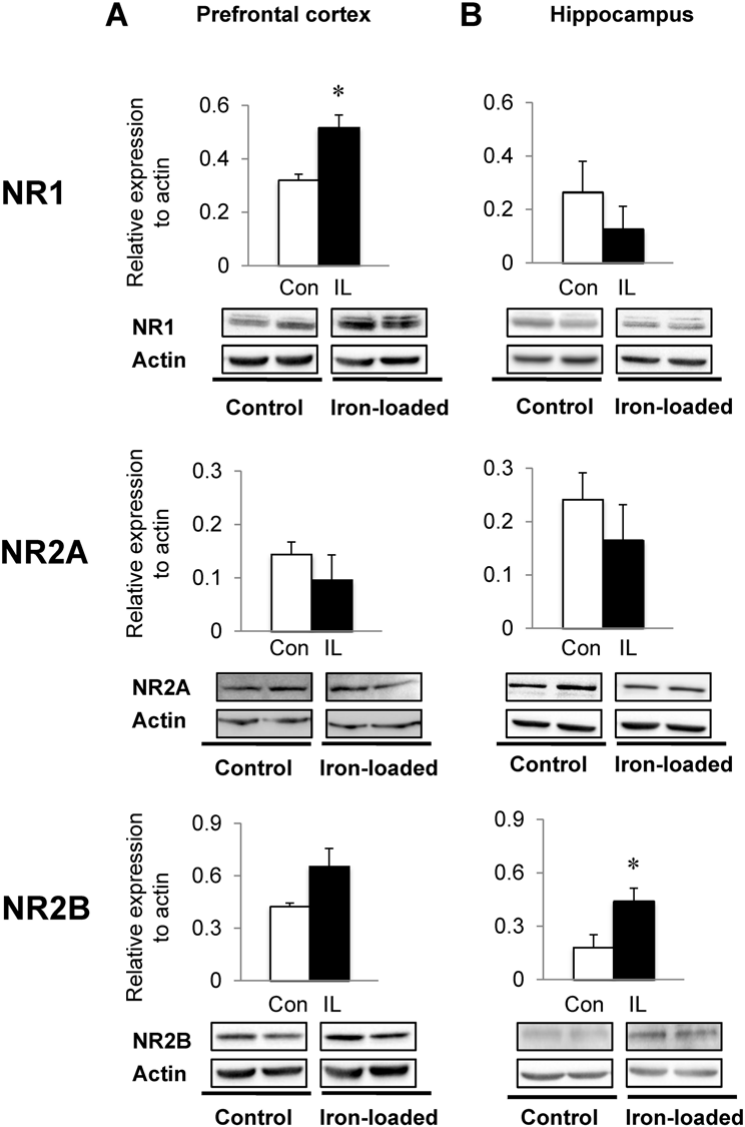
Effect of dietary iron loading on the expression of glutamate NMDA receptors in young rat brain. Prefrontal cortex (**A**) and hippocampus (**B**) were collected from rats fed iron-loading or control diet and homogenized for western blot analysis to determine the expression levels of subunits of the glutamate NMDA receptor: NR1, NR2A and NR2B. Relative intensities of protein bands normalized to actin were determined using Image Lab. Empty and closed bars represent control (Con) and iron-loaded (IL) rats, respectively. Data were presented as means ± SEM (n = 4 per group) and were analyzed using two-sample *t*-test. * *P* < 0.05.

**Fig 6:**
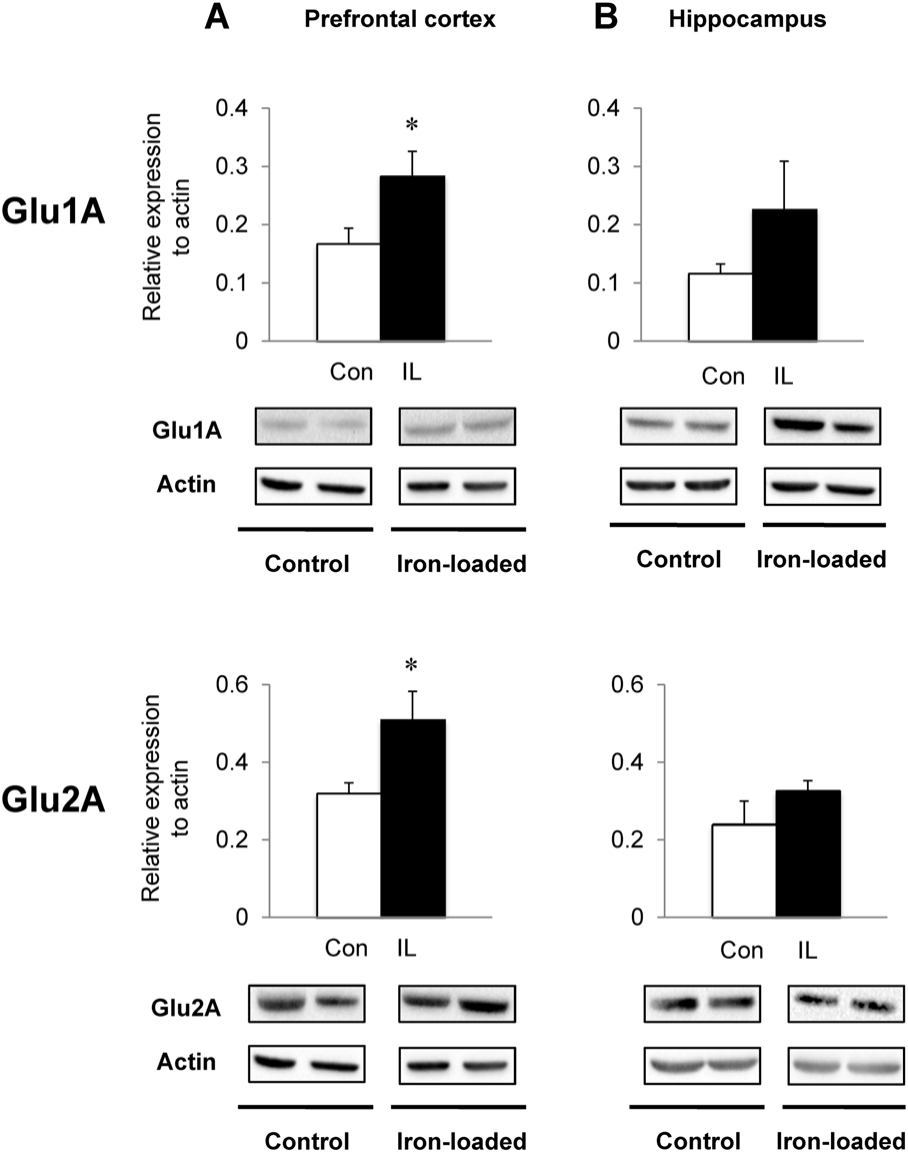
Effect of dietary iron loading on the expression of glutamate AMPA receptors in young rat brain. Prefrontal cortex (**A**) and hippocampus (**B**) were collected from rats fed iron-loading or control diet and homogenized for western blot analysis to determine the expression levels of subunits of non-NMDA ionotropic glutamate (AMPA) receptor: Glu1A and Glu2A. Relative intensities of protein bands normalized to actin were determined using Image Lab. Empty and closed bars represent control (Con) and iron-loaded (IL) rats, respectively. Data were presented as means ± SEM (n = 4 per group) and were analyzed using two-sample *t*-test. * *P* < 0.05.

**Fig 7:**
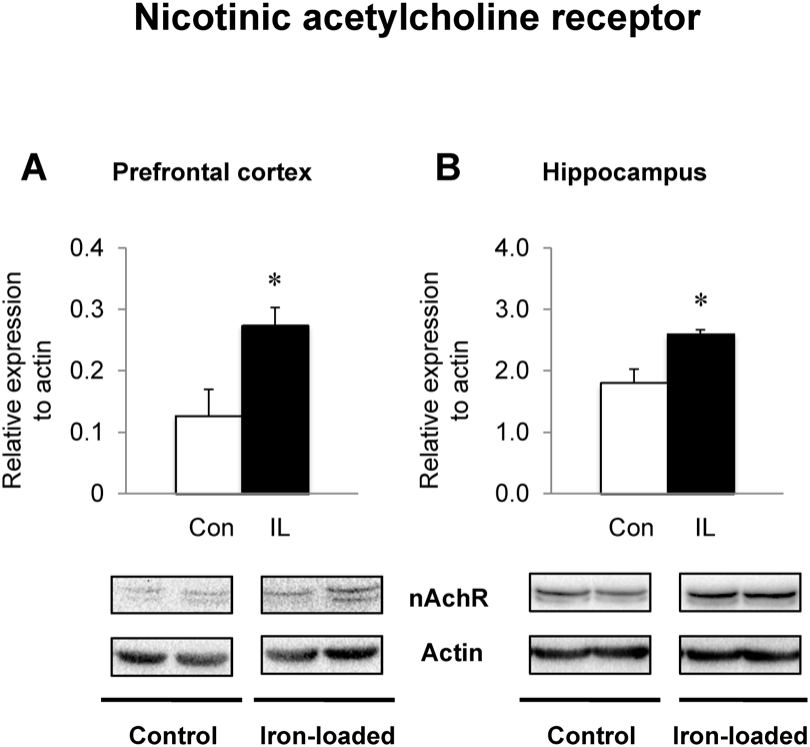
Effect of dietary iron loading on the expression of nicotinic acetylcholine receptors in young rat brain. Prefrontal cortex (**A**) and hippocampus (**B**) were collected from rats fed iron-loading or control diet and homogenized for western blot analysis to determine the expression levels of α7-nicotinic acetylcholine receptor. Relative intensities of protein bands normalized to actin were determined using Image Lab. Empty and closed bars represent control (Con) and iron-loaded (IL) rats, respectively. Data were presented as means ± SEM (n = 4 per group) and were analyzed using two-sample *t*-test. * *P* < 0.05.

Since glutamate and acetylcholine signaling is closely associated with memory performance, we examined if increased recognition memory in iron-loaded rats was due to improved glutamatergic and/or cholinergic responses in the PFC and HPC, two well-defined brain regions for memory function. The NMDA glutamate receptor NR1 subunits were up-regulated in the PFC of iron-loaded rats (62% increase; *P* = 0.009) with both NR2A and 2B unchanged (Fig. 5A), although NR2B levels trended higher (54% increase; *P* = 0.070). In the HPC from iron-loaded rats, the levels of NR1 and NR2A subunits were not altered (Fig. 5B), but NR2B levels were significantly higher in iron-loaded rats (144% increase; *P* = 0.035) compared with controls. Furthermore, the expression levels of AMPA glutamate receptors were differentially regulated in the PFC and HPC (Fig. 6); both Glu1A (70% increase; *P* = 0.042) and Glu2A (59% increase; *P* = 0.033) receptors were significantly up-regulated in the PFC of rats with high iron compared with controls, whereas the levels of these receptors were similar in the HPC between the two diet groups. In addition, iron-loaded rats showed a significant up-regulation of nAChR protein levels in the PFC compared with controls (115% increase; *P* = 0.033; Fig. 7A). Finally, the hippocampal nAChR levels were also significantly elevated in iron-loaded rats (44% increase; *P* = 0.011; Fig. 7B) compared with controls.

### Dietary iron loading does not modify oxidative stress in brain

**Fig 8:**
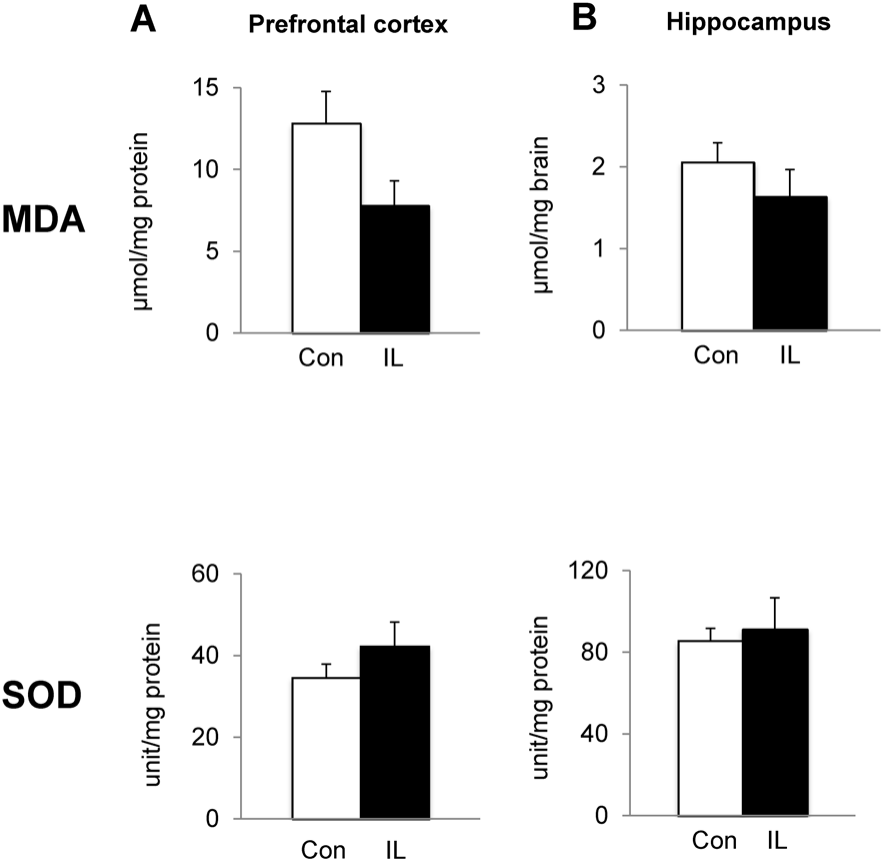
Effect of dietary iron loading on oxidative status in young rat brain. Prefrontal cortex (**A**) and hippocampus (**B**) were used to determine the levels of MDA and activities of SOD. Empty and closed bars represent control (Con) and iron-loaded (IL) rats, respectively. Data were presented as means ± SEM (n = 4 per group) and were analyzed using two-sample *t*-test.

To test if elevated iron stores increase metal-associated oxidative stress, we determined the levels of MDA, a marker of oxidative stress, and SOD, an essential anti-oxidant enzyme. In both PFC and HPC, there was no difference in either MDA levels or SOD activities between the two groups (Fig. 8).

## Discussion

A majority of investigations on iron overload in brain research have been focused on deleterious effects of iron on brain function, primarily due to iron’s ability to produce reactive oxygen species (ROS) and to promote neurodegenerative disorders, such as Alzheimer’s and Parkinson’s diseases. Experimental evidence also indicates other behavioral problems associated with oral iron loading [37,54]. Since intact BBB prevents brain from iron loading even at increased iron levels in the circulation [19,55], disruption of the BBB has been proposed as a potential mechanism of elevated brain iron and iron-mediated neurotoxicity. For example, it has been known that brain iron influx increases with the damaged BBB in some pathological conditions, such as trauma, tumor or prolonged exposure to high iron [37,54,56,57]. In particular, traumatic brain injury has been shown to be in line with increased brain iron and reduced learning capacity, which was attenuated by iron chelator deferoxamine [56,57]. More recently, Sripetchwandee *et al* [54] showed memory deficits and increased MDA levels in the brain in Wistar rats fed high iron diet for 4–8 weeks, which was associated with disruption of BBB and increased brain iron levels. In contrast, iron-loaded SD rats in our study did not show these abnormalities in the brain with MDA and SOD unchanged. This difference may be attributed to a strain-specific response to dietary iron [58]. In addition, de Lima *et al* [37] showed that postnatal oral iron administration induces impaired memory in rats, as assessed by object recognition memory task, while iron chelator corrected for loss of memory [38]. Although these results seem contradictory to our data, we note that these investigators administered ferrous iron (Fe^2+^) [37,38], which is a more reactive species than ferric iron (Fe^3+^), a much less harmful and the major form of dietary iron. It is therefore possible that a supraphysiological dose of Fe^2+^ could disrupt BBB, which is incomplete in neonates, and promote irreversible oxidative damage. Taken together, these results suggest that several factors, including different exposures (doses and routes) to iron and physiological states (e.g., age, strain, sex), could alter effects of iron loading on brain function and behavior.

We observed that iron-loaded rats significantly increased iron levels in the PFC and a similar trend was found in the hippocampus and cerebellum. However, iron stores in the striatum trended lower, although statistically insignificant, upon dietary iron loading. The striatum is vulnerable to iron-associated oxidative damage in many pathological states, including Parkinson’s disease, Alzheimer’s disease and Hungtington’s disease [59,60]. Although we do not have an immediate answer, we could speculate some mechanisms. Iron is taken up into the brain by transferrin receptor 1 (TfR1) and divalent metal transporter 1 (DMT1) and stored as a ferritin-bound form, whereas ferroportin has been proposed to export iron in order to maintain proper iron levels in the brain [61]. The regulatory mechanisms of these proteins may be different in the striatum compared with other brain regions. For example, the expression of ferritin heavy chain is increased in the cortex and hippocampus but decreased in the striatum in a model of brain ischemia [62]. Likewise, TfR1 levels are unchanged in the striatum while both cortex and hippocampus show an up-regulation of TfR1 [62]. It is also possible other transporters could be involved in striatum-specific iron transport. A recent investigation has revealed that ras homolog enriched in striatum (Rhes), a novel GTP binding protein, is activated by PKA-mediated phosphorylation and modulates iron transport *via* DMT1 in the striatum [63], and this protein is implicated in striatal pathology in Huntington’s disease [64]. Thus, increased brain iron could alter the activity of this protein in a striatum-specific manner. Finally, we cannot exclude a possibility of iron redistribution within the brain, which has been documented in iron deficiency [15,65]. It was reported that psychological stress can induce redistribution and accumulation of iron in the brain and that the striatum is relatively insensitive to iron accumulation compared with cortex and hippocampus [66,67]. A similar mechanism may operate in the striatum upon iron supplementation. The precise mechanism of region-dependent iron distribution remains to be explored.

Sobotka *et al* [39] demonstrated a dose-response relationship between dietary iron and behavioral performances with 12-wk diet regimen using weanling SD rats. Both iron-deficient rats (4 ppm iron) and rats fed extremely high iron diet (20,000 ppm) showed deficits in avoidance learning and prepulse inhibition, suggesting that any deviation from the optimal iron levels could contribute to impaired brain function. In contrast, rats fed 3,500 ppm did not exhibit considerable behavioral problems. Moreover, whole brain non-heme iron was significantly increased in animals fed 20,000 ppm iron, but not in 3,500 ppm iron [39]. This is not surprising because liver iron stores increase while brain iron stores remain relatively unchanged in adult animals overloaded with iron [68]. While 10,000 ppm iron was not tested by Sobotka *et al* [39], our data indicate that there is a preferential distribution of iron upon dietary iron loading in different brain regions; in particular, the PFC showed a significant increase in iron stores, whereas the HPC showed a trend of increase. Our results also show that increased prefrontal iron is associated with increased recognition memory function, without alterations in motor coordination or emotional behavior. It is plausible that modestly increased brain iron for a short-term period, in particular during growth, may be sufficient to provide a better memory capacity prior to producing adverse effects of metal-induced oxidative damage. This appears supported by the finding that rats fed 3,500 ppm iron diet did not show a defective behavior of prepulse inhibition until 10 weeks of iron diet [39]. Therefore, longer exposure to high dietary iron in growing animals could exhibit abnormal behavior with no benefit. Similarly, prolonged exposure to elevated systemic iron in adults who already have sufficient iron or individuals with excess iron (e.g., hemochromatosis, thalassemia and transfusional iron overload) could eventually promote brain iron loading and impair behavioral function.

Iron deficiency decreases DAT density in the striatum and nucleus accumbens [69]. Consequently, iron deficiency elevates extracellular dopamine in the caudate putamen and nucleus accumbens [69–72], which returns to normal levels when brain iron is corrected [71,72]. In contrast, DAT levels are unchanged in the PFC upon iron deficiency [45] or iron loading (present study), suggesting that iron stores do not influence DAT expression in the PFC. Alternatively, these observations may reflect brain region-specific regulation of the transporter by iron and/or differences in methods of measurement [45]. D1R has profound effects on working memory [53] and its expression is down-regulated in the caudate putamen and PFC of iron-deficient rats [73]. We here showed an up-regulation of D1R in the PFC, which was associated with increased iron concentrations in the PFC. Thus, it is possible that iron status can regulate the expression of D1R, rather than DAT, in the PFC and can modify dopamine neurotransmission pathway, which is specific to the stage of neural development. These combined results demonstrate that dopaminergic signaling pathways could be regulated by dietary iron and may contribute to recognition memory.

NMDA receptors are involved in object recognition memory; Tang *et al* demonstrated that overexpression of NR2B of the NMDA receptor complex improves object recognition memory by increasing calcium signaling [74]. A blockade of NMDA receptors by MK-801, an NMDA receptor antagonist, reduces both short- and long-term retention of object recognition memory [75]. Interestingly, Munoz *et al* have shown a beneficial effect of iron on synaptic plasticity mediated by NMDA receptors [13]. Using primary hippocampal neurons, the authors showed iron-induced ROS is necessary for increases in Ca^2+^ triggered by NMDA receptors and for long-term potentiation, which is abolished by addition of iron chelators [13]. It has been known that ROS is required for synaptic plasticity and memory [76] and NMDA receptor activation increases superoxide production through NADPH oxidase [77,78]. Moreover, glutamate and NMDA, substrates of NMDA receptors, enhance the generation of ROS [79,80]. However, we also note that hyperactivation of NMDA receptors and glutamate in excess are associated with CNS disorders and neurodegenerative diseases [81,82]. Overactivated NMDA receptors could lead to excitotoxicity by allowing large influx of Ca^2+^ into the cell and activating a variety of enzymes, such as endonuclease and phospholipase, which result in increased ROS and cell death [81,83]. In our study, although we did not measure ROS levels, there was no evidence of brain oxidative stress, as assessed by unchanged MDA and SOD, which is consistent with the finding that dietary overload does not increase whole-brain lipid peroxidation [84]. Our observation of NMDA receptor up-regulation, especially in NR1 and NR2B subunits, with a marginal increase in brain iron suggests a close link between iron and NMDA neuronal pathway within the context of memory capacity. In addition, our study showed the AMPA receptor, another type of ionotropic glutamate receptor, was elevated in the brain upon systemic iron loading, which could contribute to enhanced memory by glutamate signaling.

Acetylcholine plays another well-defined role in memory and impaired acetylcholine signaling contributes to cognitive deficits in many diseases, including Alzheimer’s disease [85,86]. Elevated cholinergic activity in the prefrontal regions could contribute to attentional and cognitive functions [87]. While elevated iron levels resulting from iron overload are associated with disruption in the cholinergic system, galantamine, a competitive inhibitor of acetylcholinesterase, has been shown to rescue iron-induced amnesia [88]. Interestingly, galantamine has been proposed to possess an activity of allosteric modulator of nAChR [89,90] and agonistic effect on NMDA receptors [91]. Consistent with this idea, our results show elevated expression levels of nAChR in iron-loaded rats. While other neurotransmitters may be involved in improved memory upon iron loading, to our knowledge, this is the first study to characterize a new molecular link between iron and nAChR expression in cognition and memory process, particularly in the prefrontal cortex and hippocampus. Further studies are warranted to determine the signaling pathways of neurotransmitters and therapeutic potential of timing of iron supplementation related to cognitive function and homeostasis of other glutamate/cholinergic receptors.
